# Prevalence and Characterization of Somatic Mutations in Chinese Aldosterone-Producing Adenoma Patients

**DOI:** 10.1097/MD.0000000000000708

**Published:** 2015-04-24

**Authors:** Baojun Wang, Xintao Li, Xu Zhang, Xin Ma, Luyao Chen, Yu Zhang, Xiangjun Lyu, Yuzhe Tang, Qingbo Huang, Yu Gao, Yang Fan, Jinzhi Ouyang

**Affiliations:** From the State Key Laboratory of Kidney Disease, Department of Urology (BW, XL, XZ, XM, LC, YZ, XL, YT, QH, YG, YF); Department of Outpatient Officer Consultation Room, PLA Medical School, Chinese People's Liberation Army General Hospital, Beijing, China (JO).

**Keywords:** aldosterone, conn adenoma, hypertension, potassium channels, sodium-potassium-exchanging ATPase

## Abstract

Recently somatic mutations of KCNJ5, ATP1A1, ATP2B3, and CACNA1D have been identified in patients with aldosterone-producing adenoma (APA). The present study sequenced the DNA in the tissues and blood samples from Chinese patients with APA for *KCNJ5*, *ATP1A1*, *ATP2B3*, and *CACNA1D* gene mutations.

Among the 114 patients, 86 (75.4%) were identified with KCNJ5 somatic mutations, including 3 previously reported (G151R, L168R, T158A) and 2 other unreported mutations. One patient presented with both a point mutation (E147) and an insertion mutation, whereas another had a 36-base duplication, G153_G164dup. No mutation of ATP1A1 and ATP2B3 in the known hotspots was identified and only 1 male patient was detected with a novel CACNA1D mutation, V748I. Unlike other studies, male and female patients had similar KCNJ5 mutation rates (76.9% vs 74.2%). Mutation carriers were younger and had lower preoperative potassium level, whereas male (but not female) mutation carriers had higher preoperative plasma aldosterone concentration and preoperative blood pressures. Mutation carriers also had higher LV mass index (LVMI) than nonmutation carriers. After surgery, LVMI improved significantly in the KCNJ5 mutation group but not in the nonmutation group. The mRNA expression of KCNJ5, CYP11B2, and ATP2B3 was higher in the KCNJ5-mutated APA tissues. Functional characterization of the 2 novel KCNJ5 mutations showed that they were associated with decreased proliferation, membrane depolarization, elevated secretion of aldosterone, and increased expression of CYP11B1 and CYP11B2.

In conclusion, Chinese APA patients appear to have a high frequency of somatic KCNJ5 mutation. Mutation prevalence rates are similar among men and women and 2 novel mutations are identified. KCNJ5-mutated patients benefit more from surgical resection of APA than nonmutated patients.

## Abstract 2

Supplemental Digital Content is available in the text

## INTRODUCTION

Primary aldosteronism (PA) is the most common cause of secondary hypertension, accounting for 4.6% to 14.4% in hypertensive patients.^1,2^ Choi et al^3^ first detected somatic and germline mutations in the *KCNJ5* gene in APAs, which encodes the potassium channel Kir 3.4 (potassium inwardly rectifying channel, subfamily J, member 5). A series of studies from different countries reported the mutation rate of KCNJ5 ranging from 30% to 60%.^4–9^ The somatic mutation identified to date includes G151R, L168R, T158A, E145Q, Ile157del, E145K, and W126R, of which G151R and L168R are the most frequent.^3,4,9–11^ The germline mutations include G151R, G151E, T158A, I157S, and Y152C.^10,12,13^ All the mutations are located in or around the selectivity filter of the K^+^ channel pore, and cause the loss of selectivity of the channel. In addition, *KCNJ5* and *CYP11B2* genes are more highly expressed in the APAs with KCNJ5 mutations than wild-type KCNJ5.^5,6,14^ Female patients have a significantly higher KCNJ5 mutation rate than male patients, which was confirmed by several studies.^4,6–9,14^ Patients with KCNJ5 mutation are significantly younger at diagnosis than those without KCNJ5 mutation.^4,7,8^ Furthermore, the KCNJ5 mutation carriers present with increased aldosterone and reduced potassium levels as previously reported.^5,8^ The adenoma size is also larger^3,4,9^ in the KCNJ5 mutation carriers.

To explore the molecular mechanisms of APA, Beuschlein et al performed exome sequencing of 9 APAs without KCNJ5 mutation, and identified 2 novel mutations in ATP1A1 (encoding an Na^+^/K^+^ ATPase α subunit) and ATP2B3 (encoding a Ca^2+^ ATPase) genes in 3 and 2 APAs, respectively.^15^ Scholl et al performed exome sequencing of 18 APAs with no KCNJ5 mutation and identified a novel gene with mutation, CACNA1D.^16^ Additional sequencing showed 5 of 43 APAs had somatic mutations in CACNA1D, including 2 mutation types, G403R and I770M. Subsequently, Azizan et al identified that 12 of 152 APAs had somatic mutations in CACNA1D.^17^

Compared with other countries, Japanese PA patients show a higher prevalence of somatic KCNJ5 mutation, with 20 of 28 APA patients carrying mutations in 2 studies, which may be attributed to the higher prevalence of APA in Japan (75% and 84.4% of PA cases in 2 studies).^18–20^ However, there are still no reported studies on somatic mutations in Chinese APA patients, and the only one among Asian subjects was relatively small. We suppose that Asian APA patients might share similar characteristics. Thus, in this study, we retrospectively analyzed 114 APAs from 2 hospitals in Beijing and Wuhan for mutations in KCNJ5, ATP1A1, ATP2B3, and *CACNA1D* genes and searched for novel mutations of these genes. In addition, we also analyzed the pre- and postoperative clinical characteristics and cardiovascular complications in the Chinese APA patients with and without *KCNJ5* gene mutations.

## MATERIALS AND METHODS

### Patients and Tumor Samples

We analyzed the medical records of 87 patients receiving surgery at Beijing PLA hospital during the period 2008 to 2013 and 27 patients receiving surgery at Wuhan Tongji hospital during 2002 to 2007 retrospectively. APA was diagnosed following previously described procedures.^21^ Written informed consent was obtained from all patients included in this study. This study was approved by the Protection of Human Subjects Committee of the Chinese PLA General Hospital. The plasma aldosterone, angiotensin II, and renin concentrations were detected by radioimmunoassay kit (Beijing North Biotechnology Research institute, China). Further details are available in the supplemental data, http://links.lww.com/MD/A241.

### DNA Sequencing of *KCNJ5*, *ATP1A1*, *ATP2B3*, and *CACNA1D* Genes

All 3 exons of *KCNJ5* gene were sequenced in tumor DNA and cDNA. Targeted sequencing of the 17 reported positions in all APA tumors were performed for *ATP1A1*, *ATP2B3*, and *CACNA1D* gene mutations. The primer sequences are given in Supplemental Table 2, http://links.lww.com/MD/A241. The PCR products were directly sequenced by GENEWIZ (Beijing, China).

### RNA Isolation, RT–PCR and Pathological Analysis

The mRNA expression of KCNJ5, CYP11B1, CYP11B2, ATP1A1, ATP2B3, and CACNA1D was quantified by RT-PCR. The primer sequences are shown in Supplemental Table 2, http://links.lww.com/MD/A241. The protein expressions of KCNJ5 and CYP11B2 were determined by immunohistochemistry. Details are shown in supplemental data, http://links.lww.com/MD/A241.

### Cell Culture, Plasmid Construction, and Transient Transfection

Cell culture and construction of the plasmid with wild-type and 2 mutant KCNJ5 sequences were described at lenth in supplemental data, http://links.lww.com/MD/A241. The insertion mutation sequence and the duplication mutation sequence were named KCNJ5-M1 and KCNJ5-M2. Subsequently, membrane voltage, cell proliferation, aldosterone secretion, and the expression of CYP11B1 and CYP11B2 were assessed, respectively. Cell proliferation was analyzed by the MTS assay (Promega, Madison, WI). The aldosterone level was detected by radioimmunoassay kit (North Institute of Biological Technology, Beijing, China). The concentration of the angiotension II for aldosterone detection was 100 nmol/L.

### Statistical Analysis

All the results are presented as mean ± standard deviation for normally distributed variables and as median (25th and 75th percentiles) for nonnormally distributed variables. The results between groups were analyzed by unpaired *t* test or analysis of variance. Nonsymmetric variables were compared using Mann–Whitney *U* test. The paired *t* test and Wilcoxon matched pairs test were used to analyze the data before and after surgery. *χ*^2^ Test was used to compare the sex ratio. Yates’ continuity corrected *χ*^2^ test was used to compare the proportion of proteinuria. All the comparisons were considered to be statistically significant at *P* < 0.05. Statistical analyses were performed using SPSS 18 (SPSS Inc, Chicago, IL).

## RESULTS

### Prevalence of Somatic APA Mutations in KCNJ5, ATP1A1, ATP2B3 and CACNA1D Genes

**FIGURE 1:**
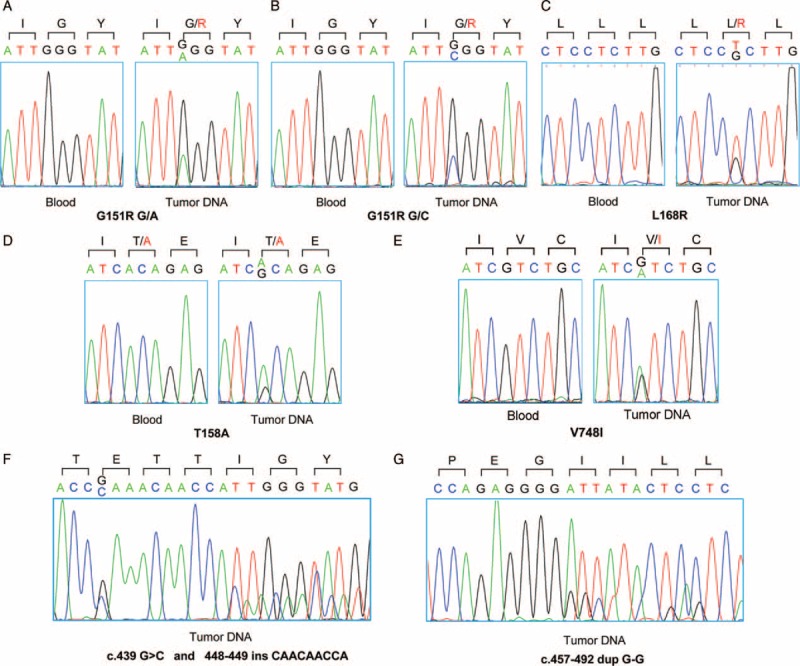
Representative sequencing chromatograms of *KCNJ5* and *CACNA1D* gene in APAs and patient blood in China. (A, B) The G151R mutation represents 2 kinds of substitutions, G151RG/C and G151RG/A, both of which result in a glycine-to-arginine change at codon 151. (C) The L168R T/G substitution leads to a glycine-to-arginine change at codon 168. (D) The T158A A/G substitution leads to a Threonine-to-Alanine change at codon 157. (E) The mutation in *CACNA1D* gene, c.2242G>A, causes a valine-to-isoleucine change at codon 748. (F) The mutations c.439G>C and c.448-449insCAACAACCA result in a glutamic acid-to-glutamine change at codon 147 and frameshift mutation at codon 150. (G) The duplication mutation c.457_492dupG_G leads to the duplication of the sequence from amino acids 153 to 164. No mutations were identified in the corresponding blood DNA samples from Beijing patients. APA = aldosterone-producing adenoma.

Of the 114 APA patients, the overall prevalence of KCNJ5 mutations in APA tissues was 75.44% (86/114) (Table 1). The p.Gly151Arg and p.Leu168Arg mutations were the most frequent, and accounted for 37.72% (43/114) and 34.21% (39/114), respectively. Of the 43 p.Gly151Arg mutations, 28 were c.451G>A and 15 were c.451G>C. Two patients presented with p.T158A mutations. In addition, 2 patients had novel somatic mutations. One patient presented with c.439 G>C and c.448–449insCAACAACCA simultaneously, resulting in a frameshift mutation, whereas another presented a duplication mutation c.457–492dupG-G. However, we failed to detect somatic mutations in the 6 previously reported affected positions of ATPases in all APA tumors. Only 1 patient had a novel CACNA1D mutation (V748I) detected. The phenotypes of the 3 patients with novel mutations were described in supplemental data, http://links.lww.com/MD/A241. No mutations were detected in all 48 available matched DNA samples from blood (Figure 1).

**TABLE 1:**
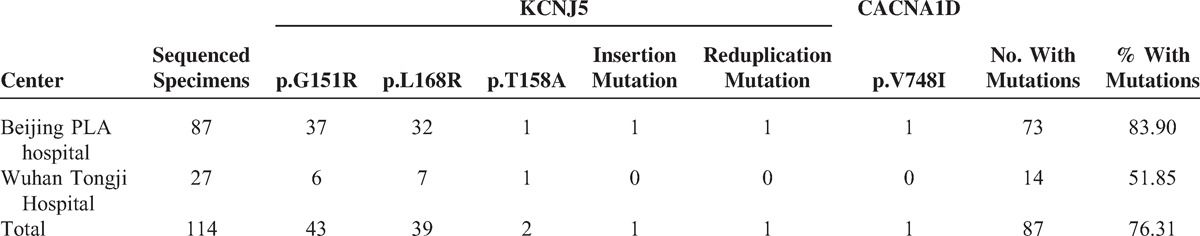
Type and Frequency of Mutations From Patients in Beijing and Wuhan

### Preoperative Clinical and Biochemical Features According to Mutation Status in Chinese APA Patients

Similar to other studies, KCNJ5 mutation carriers had younger age at diagnosis (37.6 ± 7.5 vs 43.0 ± 9.4 years), higher preoperative plasma aldosterone concentrations in recumbent position (25.1 ± 6.8 vs 18.6 ± 5.5 ng/dL) and lower preoperative potassium levels (2.90 [2.60, 3.19] vs 3.52 [3.11, 3.87] mmol/L) than patients with KCNJ5-negative APA (Table 2). Preoperative systolic blood pressures (180 [160, 190] vs 160 [150, 180] mmHg) were also higher in the mutation group. No significant differences between 2 groups were observed with regard to body mass index, preoperative aldosterone-to-renin ratio, diastolic blood pressure, adenoma size, and proteinuria. We also compared the cardiovascular parameters of 76 KCNJ5 mutation patients and 24 nonmutation patients before surgery. KCNJ5-positive APA patients had higher left ventricular mass index (LVMI) than the KCNJ5-negative APA patients (Table 2). There were no significant differences of the other parameters between the 2 groups.

**TABLE 2:**
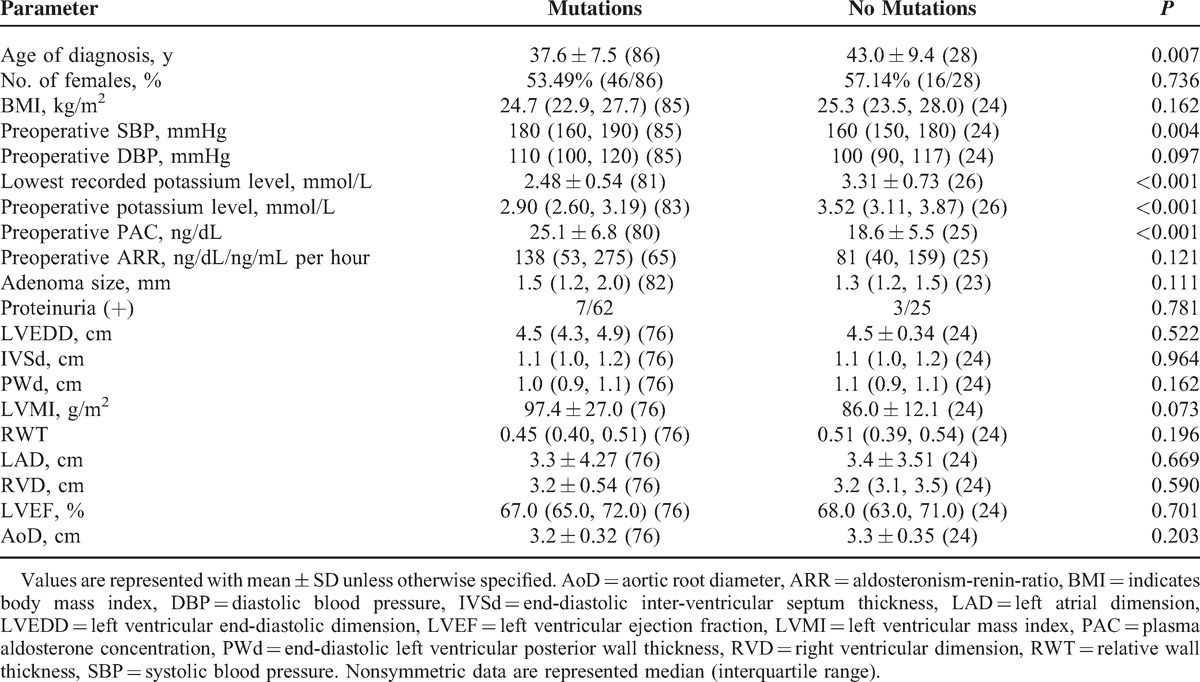
The Correlation Between *KCNJ5* Gene Mutation Status and Preoperative Clinical and Biochemical Data

### Prevalence of KCNJ5 Mutations and Clinical Features According to Sex

There were no significant differences in the prevalence of KCNJ5 mutations between Chinese men and women (76.9% [40/53] vs 74.2% [46/62], *P* = 0.736) (Supplemental Table 3, http://links.lww.com/MD/A241). Males with KCNJ5 mutations presented with significantly higher preoperative systolic (180 [163, 198] vs 150.0 [140, 165] mmHg, *P* = 0.0005), diastolic blood pressures (110 [110, 120] vs 100.0 [93, 105] mmHg, *P* = 0.0054) and preoperative plasma aldosterone concentration in recumbent position (26.1 ± 7.4 vs. 14.5 ± 5.0, *P* < 0.0001), whereas these differences were absent in female patients (Supplemental Table 3, http://links.lww.com/MD/A241).

### Postoperative Clinical and Biochemical Features According to Mutation Status in Chinese APA Patients

Table 3 showed that systolic blood pressure, diastolic blood pressure, and plasma aldosterone concentration decreased, whereas the serum potassium level increased significantly after surgery. Although there were no significant differences of these postoperative parameters between 2 groups, the proportion of positive proteinuria was also similar between the 2 groups. The LVMI, left ventriclar end-diastolic dimension, end-diastolic inter-ventricular septum thickness, and left atrial dimension improved significantly after surgery in the KCNJ5 mutation group but not in the KCNJ5 nonmutation group. No significant differences were observed between the 2 groups for these postoperative cardiovascular parameters.

**TABLE 3:**
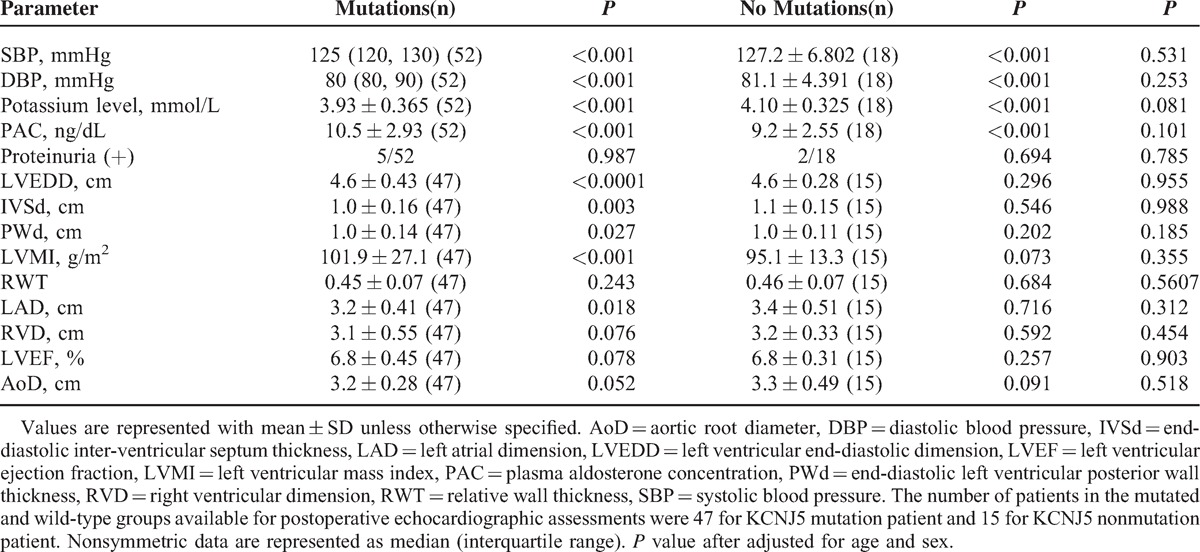
The Correlation Between *KCNJ5* Gene Mutation Status and Postoperative Clinical and Biochemical Data

### Gene Expression Analysis in APA With Different Mutation Types

**FIGURE 2:**
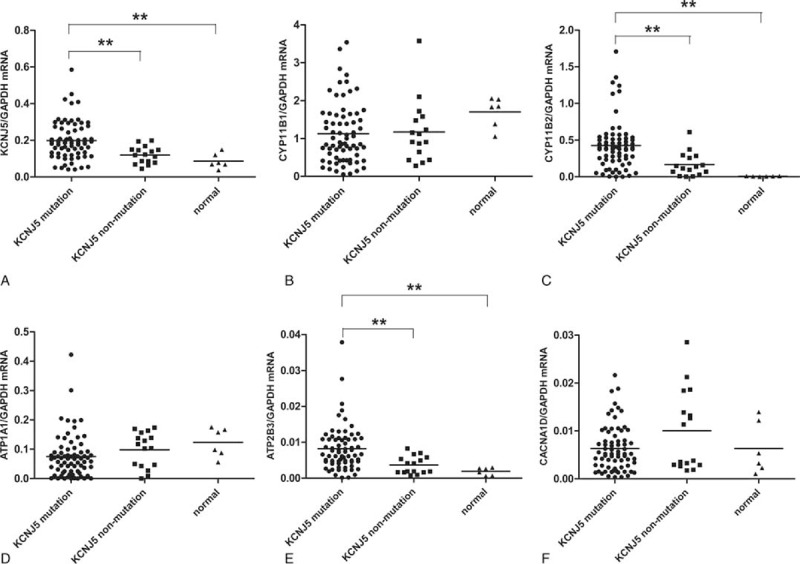
mRNA expression levels of *KCNJ5*, *CYP11B1*, *CYP11B2*, *ATP1A1*, *ATP2B3*, and *CACNA1D* genes in 87 APAs from Beijing grouped for KCNJ5 mutation status and 6 normal adrenal glands. (A) mRNA expression levels of *KCNJ5* gene in 87 APAs from Beijing grouped for KCNJ5 mutation status. (B) mRNA expression levels of *CYP11B1* gene in 87 APAs from Beijing grouped for KCNJ5 mutation status.(C) mRNA expression levels of *CYP11B2* gene in 87 APAs from Beijing grouped for KCNJ5 mutation status. (D) mRNA expression levels of *ATP1A1* gene in 87 APAs from Beijing grouped for KCNJ5 mutation status. (E) mRNA expression levels of *ATP2B3* gene in 87 APAs from Beijing grouped for KCNJ5 mutation status. (F) mRNA expression levels of *CACNA1D* gene in 87 APAs from Beijing grouped for KCNJ5 mutation status. The KCNJ5, CYP11B2, and ATP2B3 mRNA expression levels were significantly higher in APA with KCNJ5 mutations (*n* = 71) than those without KCNJ5 mutations (*n* = 16). The KCNJ5, CYP11B2, and ATP2B3 mRNA expression levels were significantly lower in 6 normal adrenal gland tissue compared with tumors. Each bar represents the mean ± SE of relative gene expression in 3 independent experiments. The relative mRNA levels were normalized to glyceraldehyde-phosphate dehydrogenase (GAPDH) using the 2^−ΔΔCT^ method. The median of each group and *P* value are indicated in the graph. APA = aldosterone-producing adenoma.

**FIGURE 3:**
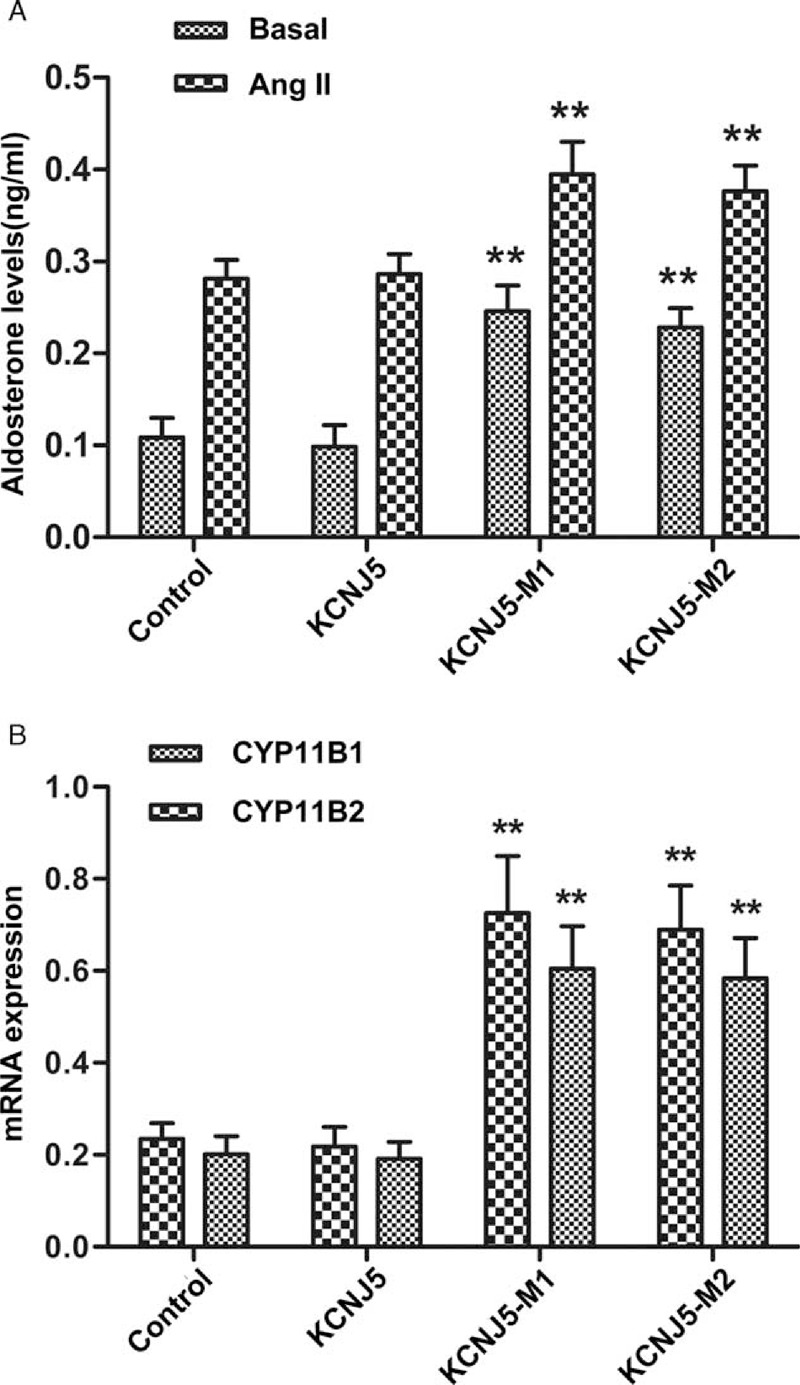
Effect of KCNJ5-M1 and KCNJ5-M2 on the aldosterone secretion and mRNA expression of CYP11B1 and CYP11B2. (A) Basal and A-II stimulated secretion of aldosterone production by H295R cells transfected with control, wild-type KCNJ5, KCNJ5-M1, and KCNJ5-M2 plasmids. Cells were incubated for 24 hours in the absence and presence of 100 nmol/L A-II after 24 hours of transfection. ^∗∗^*P* < 0.01, *n* = 5. (B) CYP11B1 and CYP11B2 mRNA expression levels in the H295R cells 24 hours after transfection with control, wild-type, and mutant KCNJ5 plasmids. Each bar represents the mean ± SE of relative gene expression in 3 independent experiments. The mRNA levels were normalized to glyceraldehyde-phosphate dehydrogenase (GAPDH) using the 2^−ΔΔCT^ method. ^∗∗^*P* < 0.01.

We measured the mRNA expression of KCNJ5, CYP11B1, CYP11B2, ATP1A1, ATP2B3, and CACNA1D using real-time PCR. Patients with KCNJ5 mutation had a significantly higher mRNA expression of KCNJ5, CYP11B2, and ATP2B3 than those without KCNJ5 mutation (Figure 2; *P* = 0.0004 and *P* = 0.0002, respectively). The 3 genes also exhibited a significantly lower expression in the normal adrenal gland compared with those in tumors. We also detect the KCNJ5 and CYP11B2 at protein level in different KCNJ5 mutation type by immunohistochemistry. KCNJ5 staining was observed on the membrane of almost all cells in KCNJ5 nonmutant APA. Every type of KCNJ5 mutant APA showed increased staining of KCNJ5. Accordingly, CYP11B2 staining was also slightly strong in mutant APA compared with nonmutant APA (Supplemental Figure 1, http://links.lww.com/MD/A241) (Figure 3).

### Functional Effects of the Insertion and Duplication Mutations

After transfection for 36 hours, HEK293 cells transfected with KCNJ5-M1 and KCNJ5-M2 exhibited increased intensity of fluorescence in more cells compared with control group (Supplemental Figure 2, http://links.lww.com/MD/A241). These results showed that both the 2 novel mutations can depolarize the HEK293 cell membrane potential. The growth curves demonstrated that HEK293 cells transfected with KCNJ5-M1 and KCNJ5-M2 showed decreased proliferation after 72 hours. The mean absorbance at 490 nm of KCNJ5-M1 and KCNJ5-M2 were both lower than the KCNJ5 and control group at 96^th^ hour (2.2 ± 0.15 for M1, 2.3 ± 0.14 for M2, 2.9 ± 0.13 for KCNJ5 and 2.98 ± 0.14 for control) (Supplemental Figure 2, http://links.lww.com/MD/A241).

Transfection of the H295R cells with plasmid carrying the KCNJ5-M1 and KCNJ5-M2 sequences increased the basal aldosterone production by 2.27- and 2.10-fold over the control cells, respectively, whereas cells transfected with the wild-type KCNJ5 slightly decreased aldosterone production. To compare the effects of the 2 mutations on aldosterone production induced by A-II, we compared the aldosterone production of supernatants after stimulation with A-II for 24 hours. The result showed that aldosterone production in the KCNJ5-M1 and KCNJ5-M2 mutation groups increased by 1.40- and 1.34-fold than that in the control H295R cells. The KCNJ5-M1-transfected cells showed a 3.00- and 3.09-fold increase in the mRNA expression of CYP11B1 and CYP11B2, whereas KCNJ5-M2-transfected cells showed a 2.90- and 2.94-fold increase in the mRNA expression of the 2 genes.

## DISCUSSION

In the present study, we show that Chinese patients with APA presented a higher prevalence of mutation of the *KCNJ5* gene. The similarity of frequency with the Japanese population reveals that the mutation rate of the *KCNJ5* gene may have racial discrepancies. However, reports by Sang et al, Loh et al, and Sy et al,^22–24^ respectively showed that about 55.7, 50, and 48.7% of Chinese PA patients had APA. This is lower than that in Japanese PA patients (75% and 84.4% of PA cases in 2 studies).^18–20^ Therefore, the high prevalence of KCNJ5 mutation in Chinese PA patients could not be attributed to the proportion of APA patients. Notably, in 2 studies on European patients,^4,8^ KCNJ5 mutations were far more frequent in women than those in men (49% vs 19% and 63% vs 24%), but a significant difference was not observed in the Chinese male and female patients (76.9% [40/52] vs 74.2% [46/62], *P* = 0.736). So the higher mutation frequency could be partially attributed to the higher frequency in male PA patients.

We did not identify any case with mutations in the 6 hotspots of *ATP1A1* and *ATP2B3* genes. Only one novel CACNA1D mutation, namely V748I, in 1 patient was identified. Interestingly, the patient was an older male presenting with a mild PA and his tumor was 2.0 × 1.5 × 0.7 cm. His blood pressure could be controlled under medication with calcium channel blockers. With the exception of the tumor size, all of these clinical characteristics were consistent with those reported previously for patients with somatic CACNA1D mutations.^16^

Funder^25^ suggested that, typically, 50% of APAs were <10 mm and 50% were associated with hypokalemia. In our study, 90 patients (78.95%) had hypokalemia, and 29 patients (27.6%) had a tumor >20 mm in diameter with an overall mean diameter of 15.49 mm. Thus, the subjects in the present study may have severe APA. The two hospitals involved are both the top ones in the province, leading to relative severely manifested APA patients in our study. APA patients from Beijing had a higher KCNJ5 mutation rate (82.76%) than patients from Wuhan (51.85%) (shown in Supplemental Table 1, http://links.lww.com/MD/A241). Correspondingly, patients from Beijing presented with higher preoperative plasma aldosterone concentration and lower plasma potassium level than those from Wuhan. In addition, the age at diagnosis is also slightly younger in Beijing patients. So the more severe manifestation of the patients from Beijing may be the main reason accounting for the mutation discrepancy of 2 centers. Second, patients from Wuhan hospital most live in southern China, whereas patients from PLA General Hospital most live in northern China. The habit of higher salt intake in northern China may partially explain the lower plasma K^+^ levels from Beijing.

As previously reported, the KCNJ5 mutation carriers display younger age at diagnosis, higher preoperative plasma aldosterone concentration, and lower preoperative potassium levels compared with the KCNJ5-negative APA cases.^4,5,8,9^ Males, but not females, with KCNJ5 mutations had significantly higher preoperative systolic, diastolic blood pressures, and preoperative plasma aldosterone levels in recumbent position than those without mutations. Therefore, we supposed that the mutation of *KCNJ5* gene might exert a greater effect on the production of aldosterone manifestation of Chinese male patients. Other studies also suggested that male APA patients might have severe phenotype and more complications,^26,27^ which may be explained by the protecting effect of estrogens and progesterones in female patients.^28^

Consistent with a previous study,^29^ KCNJ5-mutated APA patients have higher LVMI value than KCNJ5 nonmutated patients, although there was no significance in our study. KCNJ5 mutations cause more marked hyperaldosteronism, combination of another two studies,^29,30^ KCNJ5-mutated patients suffer from more cardiovascular complications than KCNJ5 nonmutated ones. However, data about renal damage are still lacking. LVMI was significantly reduced in the KCNJ5-mutated APA patients but not in the nonmutated patients after surgery. These results suggested that KCNJ5-mutated patients benefit more from surgical resection of APA than nonmutated patients.

Both KCNJ5 and CYP11B2 had elevated mRNA expression levels in the KCNJ5 mutation group, which was consistent with other studies,^5,6,31^ whereas inconsistent with another study with large sample.^7^ Mutant APA sections had also intense staining of KCNJ5 protein. The CYP11B2 staining is only slightly strong in the KCNJ5 mutant sections. This is inconsistent with other studies showing that KCNJ5-mutant APAs have a lower expression of CYP11B2 and KCNJ5.^17^ We speculate that this may attribute to the heterogeneity of nonmutant APA section. Similarly, ATP2B3 had a higher mRNA expression in the mutated tissues. We speculate that more plasma membrane Ca^2+^ ATPase (encoded by ATP2B3) is required to sustain intracellular calcium homeostasis in the KCNJ5-mutated cells, which may lead to the elevated expression of ATP2B3. The expression of the 3 genes above was also lower in the normal adrenal gland. Immunohistology staining also showed increased expression of KCNJ5 and CYP11B2 in the KCNJ5 mutation tissues. Our result of KCNJ5 staining is consistent with the study from Japan in that increased staining of KCNJ5 is observed in KCNJ5 mutant APA.^6^ Azizan et al^17^ report that KCNJ5 mutations are common in APAs resembling cortisol-secreting cells of adrenal zona fasciculata. However, the CYP11B2 staining in our study is increased in the KCNJ5 mutant APAs, which is consistent with the mRNA expression. Therefore, we suppose that higher expression of CYP11B2 protein results in the increased aldosterone and higher blood pressure of APA patients.

We also identified 2 novel somatic KCNJ5 mutations. One mutation resulted in point mutation E147Q and frameshift mutation at codon 150. E147Q has been shown to have minimal effects on Kir3.4 ion selectivity,^32^ whereas a frameshift mutation can severely damage the GYG motif. Another mutation resulted in the duplication of the sequence from amino acids 153 to 164, which prolonged the protein sequence near the GYG motif and possibly affected K^+^ selectivity. Functional tests showed that HEK293 cells transfected with the 2 mutant KCNJ5 sequences had more DiSBAC_2_ in cells, indicating higher membrane voltage of transfected cells (Supplemental Figure 2, http://links.lww.com/MD/A241). These cells also proliferated poorly, which may be caused by severe membrane depolarization. The mRNA expression of CYP11B1 and CYP11B2 and aldosterone production also increased in H295R cells transfected with the 2 mutant K^+^ channels. Like other reported KCNJ5 mutation type, this showed that the 2 mutations could increase the expressions of cortisol and aldosterone synthesizing related genes. The novel CACNA1D mutation, V748I, is located between the 2 previously reported mutations, F747L and I750M. It may affect the channel activation gate in the second domain.

However, the limitations remain in our study. Only 97 of the 114 patients were followed up for postoperative data. Considering the missing data of the 17 patients, our conclusion is not so determined. The prognosis between the KCNJ5 mutation group and nonmutation group still needs further exploration. Second, we did not obtain enough samples from the patients in Wuhan Tongji hospital for RNA extraction, so the determination of mRNA expression was completely from samples in Beijing PLA hospital. Finally, we only obtained the tumor samples that were resectable and recognizable from the patients receiving operations in the 2 hospitals, so the selection bias and the admission bias may lead to overestimation of the mutation frequency.

In summary, we identified a higher prevalence of KCNJ5 mutation in Chinese APA patients than that in other countries, and 1 patient with CACNA1D mutation, but no mutation was observed in the 6 hotspots of *ATP1A1* and *ATP2B3* genes. Chinese male APA patients presented a similar mutation rate to females. Also, KCNJ5-mutated patients benefit more from surgical resection of APA than nonmutated patients. We identified 2 novel somatic KCNJ5 mutation types and one novel CACNA1D mutation, V748I. These findings suggest that Chinese APA patients may have different genetic characteristics to those of patients from other countries.

## References

[1] Funder JW Carey RM Fardella C Case detection, diagnosis, and treatment of patients with primary aldosteronism: an endocrine society clinical practice guideline. *J Clin Endocrinol Metab* 2008; 93:3266–3281. 18552288 10.1210/jc.2008-0104

[2] Plouin PF Amar L Chatellier G Trends in the prevalence of primary aldosteronism, aldosterone-producing adenomas, and surgically correctable aldosterone-dependent hypertension. *Nephrol Dial Transplant* 2004; 19:774–777. 15031328 10.1093/ndt/gfh112

[3] Choi M Scholl UI Yue P K+ channel mutations in adrenal aldosterone-producing adenomas and hereditary hypertension. *Science* 2011; 331:768–772. 21311022 10.1126/science.1198785 PMC3371087

[4] Akerstrom T Crona J Delgado Verdugo A Comprehensive re-sequencing of adrenal aldosterone producing lesions reveal three somatic mutations near the KCNJ5 potassium channel selectivity filter. *PloS One* 2012; 7:e41926. 22848660 10.1371/journal.pone.0041926 PMC3407065

[5] Monticone S Hattangady NG Nishimoto K Effect of KCNJ5 mutations on gene expression in aldosterone-producing adenomas and adrenocortical cells. *J Clin Endocrinol Metab* 2012; 97:E1567–1572. 22628608 10.1210/jc.2011-3132 PMC3410264

[6] Taguchi R Yamada M Nakajima Y Expression and mutations of KCNJ5 mRNA in Japanese patients with aldosterone-producing adenomas. *J Clin Endocrinol Metab* 2012; 97:1311–1319. 22278422 10.1210/jc.2011-2885

[7] Fernandes-Rosa FL Williams TA Riester A Genetic spectrum and clinical correlates of somatic mutations in aldosterone-producing adenoma. *Hypertension* 2014; 64:354–361. 24866132 10.1161/HYPERTENSIONAHA.114.03419

[8] Boulkroun S Beuschlein F Rossi GP Prevalence, clinical, and molecular correlates of KCNJ5 mutations in primary aldosteronism. *Hypertension* 2012; 59:592–598. 22275527 10.1161/HYPERTENSIONAHA.111.186478

[9] Azizan EA Murthy M Stowasser M Somatic mutations affecting the selectivity filter of KCNJ5 are frequent in 2 large unselected collections of adrenal aldosteronomas. *Hypertension* 2012; 59:587–591. 22252394 10.1161/HYPERTENSIONAHA.111.186239

[10] Scholl UI Nelson-Williams C Yue P Hypertension with or without adrenal hyperplasia due to different inherited mutations in the potassium channel KCNJ5. *Proc Natl Acad Sci U S A* 2012; 109:2533–2538. 22308486 10.1073/pnas.1121407109 PMC3289329

[11] Williams TA Monticone S Schack VR Somatic ATP1A1, ATP2B3, and KCNJ5 mutations in aldosterone-producing adenomas. *Hypertension* 2014; 63:188–195. 24082052 10.1161/HYPERTENSIONAHA.113.01733

[12] Mulatero P A new form of hereditary primary aldosteronism: familial hyperaldosteronism type III. *J Clin Endocrinol Metab* 2008; 93:2972–2974. 18685118 10.1210/jc.2008-1241

[13] Monticone S Hattangady NG Penton D a Novel Y152C KCNJ5 mutation responsible for familial hyperaldosteronism type III. *J Clin Endocrinol Metab* 2013; 98:E1861–E1865. 24037882 10.1210/jc.2013-2428 PMC3816265

[14] Williams TA Monticone S Crudo V Visinin-like 1 is upregulated in aldosterone-producing adenomas with KCNJ5 mutations and protects from calcium-induced apoptosis. *Hypertension* 2012; 59:833–839. 22331379 10.1161/HYPERTENSIONAHA.111.188532

[15] Beuschlein F Boulkroun S Osswald A Somatic mutations in ATP1A1 and ATP2B3 lead to aldosterone-producing adenomas and secondary hypertension. *Nat Genet* 2013; 45:440–444.444e441–442. 23416519 10.1038/ng.2550

[16] Scholl UI Goh G Stolting G Somatic and germline CACNA1D calcium channel mutations in aldosterone-producing adenomas and primary aldosteronism. *Nat Genet* 2013; 45:1050–1054. 23913001 10.1038/ng.2695 PMC3876926

[17] Azizan EA Poulsen H Tuluc P Somatic mutations in ATP1A1 and CACNA1D underlie a common subtype of adrenal hypertension. *Nat Genet* 2013; 45:1055–1060. 23913004 10.1038/ng.2716

[18] Omura M Sasano H Saito J Clinical characteristics of aldosterone-producing microadenoma, macroadenoma, and idiopathic hyperaldosteronism in 93 patients with primary aldosteronism. *Hypertens Res* 2006; 29:883–889. 17345788 10.1291/hypres.29.883

[19] Nishikawa T Saito J Omura M Prevalence of primary aldosteronism: should we screen for primary aldosteronism before treating hypertensive patients with medication? *Endocr J* 2007; 54:487–495. 17124364 10.1507/endocrj.kr-105

[20] Omura M Saito J Yamaguchi K Prospective study on the prevalence of secondary hypertension among hypertensive patients visiting a general outpatient clinic in Japan. *Hypertens Res* 2004; 27:193–202. 15080378 10.1291/hypres.27.193

[21] Wang B Zhang G Ouyang J Association of DNA polymorphisms within the CYP11B2/CYP11B1 locus and postoperative hypertension risk in the patients with aldosterone-producing adenomas. *Urology* 2010; 76: 1018 e1011–e1017. 10.1016/j.urology.2010.03.019 20708777

[22] Sang X Jiang Y Wang W Prevalence of and risk factors for primary aldosteronism among patients with resistant hypertension in China. *J Hypertens* 2013; 31:1465–1471.discussion 1471–1462. 24006040 10.1097/HJH.0b013e328360ddf6

[23] Loh KC Koay ES Khaw MC Prevalence of primary aldosteronism among Asian hypertensive patients in Singapore. *J Clin Endocrinol Metab* 2000; 85:2854–2859. 10946893 10.1210/jcem.85.8.6752

[24] Sy WM Fu SN Luk W Primary hyperaldosteronism among Chinese hypertensive patients: how are we doing in a local district in Hong Kong. *Hong Kong Med J* 2012; 18:193–200. 22665682

[25] Funder JW The genetic basis of primary aldosteronism. *Curr Hypertens Rep* 2012; 14:120–124. 22359160 10.1007/s11906-012-0255-x

[26] Lu ZH Zhu XX Tang ZQ Female sex hormones are associated with the reduction of serum sodium and hypertension complications in patients with aldosterone-producing adenoma. *Endocr J* 2013; 60:1261–1268. 24018882 10.1507/endocrj.ej13-0123

[27] Stowasser M Bachmann AW Huggard PR Severity of hypertension in familial hyperaldosteronism type I: relationship to gender and degree of biochemical disturbance. *J Clin Endocrinol Metab* 2000; 85:2160–2166. 10852446 10.1210/jcem.85.6.6651

[28] Boschitsch E Mayerhofer S Magometschnigg D Hypertension in women: the role of progesterone and aldosterone. *Climacteric* 2010; 13:307–313. 20443718 10.3109/13697131003624649

[29] Rossi GP Cesari M Letizia C KCNJ5 gene somatic mutations affect cardiac remodelling but do not preclude cure of high blood pressure and regression of left ventricular hypertrophy in primary aldosteronism. *J Hypertens* 2014; 32:1514–1521.discussion 1522. 24759126 10.1097/HJH.0000000000000186

[30] Kitamoto T Suematsu S Matsuzawa Y Comparison of cardiovascular complications in patients with and without KCNJ5 gene mutations harboring aldosterone-producing adenomas. *J Atheroscler Thromb* 2015; 22:191–200. 25253161 10.5551/jat.24455

[31] Azizan EA Lam BY Newhouse SJ Microarray, qPCR, and KCNJ5 sequencing of aldosterone-producing adenomas reveal differences in genotype and phenotype between zona glomerulosa- and zona fasciculata-like tumors. *J Clin Endocrinol Metab* 2012; 97:E819–829. 22442279 10.1210/jc.2011-2965

[32] Dibb KM Rose T Makary SY Molecular basis of ion selectivity, block, and rectification of the inward rectifier Kir3.1/Kir3.4 K(+) channel. *J Biol Chem* 2003; 278:49537–49548. 14504281 10.1074/jbc.M307723200

